# Age Dependence of Immunity Induced by a Candidate Universal Influenza Vaccine in Mice

**DOI:** 10.1371/journal.pone.0153195

**Published:** 2016-04-07

**Authors:** Mayra García, Julia A. Misplon, Graeme E. Price, Chia-Yun Lo, Suzanne L. Epstein

**Affiliations:** Division of Cellular and Gene Therapies, Center for Biologics Evaluation and Research, Food and Drug Administration, Silver Spring, Maryland, United States of America; University of Iowa, UNITED STATES

## Abstract

Influenza has a major impact on the elderly due to increased susceptibility to infection with age and poor response to current vaccines. We have studied universal influenza vaccine candidates based on influenza A nucleoprotein and matrix 2 (A/NP+M2). Long-lasting protection against influenza virus strains of divergent subtypes is induced, especially with mucosal immunization. Here, we tested universal vaccination in BALB/c mice of different ages. Vaccination used intramuscular DNA priming to A/NP+M2 followed by intranasal (i.n.) boosting with recombinant adenoviruses (rAd) expressing the same antigens, or only A/NP+M2-rAd given i.n. Antigen-specific systemic antibody responses were induced in young, middle-aged, and elderly mice (2, 11–17, and 20 months old, respectively), but decreased with age. Antibody responses in bronchoalveolar lavage (BAL) were detected only in young mice. Antigen-specific T cell responses were seen in young and middle-aged but not elderly mice. A/NP+M2 vaccination by the two regimens above protected against stringent challenge in young and middle-aged mice, but not in elderly mice. However, mice vaccinated with A/NP-rAd or A/M2-rAd during their youth were partially protected against challenge 16 months later when they were elderly. In addition, a regimen of two doses of A/NP+M2-rAd given i.n. one month apart beginning in old age protected elderly mice against stringent challenge. This study highlights the potential benefit of cross-protective vaccines through middle age, and suggests that their performance might be enhanced in elderly individuals who had been exposed to influenza antigens early in life, as most humans have been, or by a two-dose rAd regimen given later in life.

## Introduction

Influenza poses a public health threat, especially to the very young, elderly, or chronically ill. Influenza killed about 10,000–20,000 people 65 years and older in the US alone in 2006–2007 [1]. Lower vaccine efficacy [2, 3] due to reduced immune function and underlying illnesses may contribute to susceptibility of the elderly. Conventional influenza vaccines are poorly immunogenic in the elderly [4], with at least 20% of elderly recipients failing to develop protective hemagglutination inhibition (HAI) antibody titers after vaccination [5]. Given the increasing elderly population, it is critical to evaluate how the senescent immune system responds to vaccines, and to develop strategies to improve vaccine effectiveness [6, 7].

Vaccination is a valuable public health strategy for controlling influenza. However, viral mutation and reassortment frequently alter the neutralizing antibody targets hemagglutinin (HA) and neuraminidase (NA), rendering existing vaccines ineffective. Preparation of updated strain-matched vaccines takes months [8]. An attractive alternative would be universal influenza vaccines inducing heterosubtypic immunity against relatively conserved influenza proteins. Such vaccines would protect against diverse influenza virus strains of all subtypes (reviewed in [9]). However, vaccines targeting conserved antigens do not induce neutralizing antibodies and so do not prevent infection. Instead they limit disease severity, viral replication and shedding [9], and transmission [10]. Universal vaccine candidates have been based on M2 [11], NP [12], NP+M2 [13], M1 [14], HA-stem [15] and attenuated influenza viruses [16, 17].

We have shown that DNA prime-recombinant adenovirus (rAd) boost vaccination to highly conserved influenza antigens A/NP+M2 induces effective cross-protective immunity against H1N1, H3N2, and H5N1 challenges [13, 18]. We subsequently showed that single-dose rAd was effective with or without DNA priming. In mice, rAd was more effective when given intranasally (i.n.) than intramuscularly (i.m.), resulting in increased survival, decreased morbidity, and reduced lung viral titers [19]. Increased virus-specific IgA responses and stronger lung T cell responses were induced by i.n. rAd and likely contributed to its effectiveness [19].

Because cellular immune responses are decreased in the elderly, it is important to assess the capacity of cross-protective vaccines which utilize not only antibody but also cellular responses to protect the aged population. Therefore, we tested A/NP+M2 vaccination in mice 2, 11–17, and 20 months old, approximating young (˂20 years old), middle-aged (40–54 years old), and elderly (roughly 56–69 years old) humans respectively [20]. We measured antibody and T cell responses in systemic and mucosal samples, and protection against challenge, and explored additional vaccination regimens with potential to protect the elderly.

## Materials and Methods

### Vectors

CMV/R-based plasmids expressing NP (A/NP) from A/PR/8/34 (H1N1) or M2 of the consensus sequence widely expressed in human influenza virus strains (A/M2Con, called M2 here), or NP from influenza B/Ann Arbor/1/86 (B/NP) have been described [21–23]. rAd vectors (Ad5-ΔE1ΔE3) expressing A/NP or A/M2Con, or B/NP have been described [23, 24]. All rAd stocks were prepared, tested for replication competence, and particle titers calculated from OD_260_ by ViraQuest, Inc. and stored in A195 buffer/5% sucrose. pAd⁄CMV⁄V5-GW/lacZ, a replication-incompetent Ad5 vector expressing lacZ, was constructed using Gateway cloning and the ViraPower Adenoviral Expression System (Invitrogen, Carlsbad, CA) by previously described methods [23]. The insert was derived from the control lacZ plasmid in the kit.

### Viruses

Mouse-adapted A/FM/1/47-ma (H1N1) [A/FM-ma] [25] was obtained from Earl Brown, University of Ottawa, and prepared as described [13]. A/Mexico/4108/2009 (H1N1) [A/Mex], kindly provided by Hang Xie and Zhiping Ye, CBER, Food and Drug Administration, was grown in MDCK cells (ATCC, Manassas, VA) in Opti-MEM with 1 μg/ml TPCK-trypsin (Worthington Biochemical, Lakewood, NJ). Supernatant was harvested at 90% cytopathic effect, spun 3 times at 304 *g* and supplemented with 3% (final) bovine serum albumin (Fisher BioReagents, Fair Lawn, NJ). Stocks were frozen at -80°C. 50% tissue culture infectious doses (TCID_50_) [26] were determined by the method of moving averages [27, 28]. Challenge doses of the A/FM-ma stock in TCID_50_ units are given in figure legends. Influenza A/FM-ma virus and A/Mexico virus, as well as the recombinant adenoviruses NP-rAd and M2-rAd, were tested and contaminants ruled out by a standard screening for mouse pathogens (IMPACT II PCR Profile), by RADIL (later named IDEXX RADIL), Columbia, MD.

### Mice

Female BALB/cAnNCr mice 4 weeks old were purchased from the National Cancer Institute, Frederick, MD and rested until use. Female BALB/cBy mice 20 months old were purchased from the National Institute on Aging/Charles River (Kingston, NY). BALB/cJ animals from Jackson Laboratories were kindly provided by Maryna Eichelberger, CBER/FDA at 11 months of age and rested until use. BALB/cBy and BALB/cJ mice 4 weeks old were bought from Jackson Laboratories (Bar Harbor, ME). Use of multiple BALB/c sublines was necessitated by difficulties obtaining aged mice. Each experiment used young and old mice of matched subline and supplier to control for genetic effects.

### Mouse immunizations and challenge infections

DNA priming used 50 μg each of A/NP and A/M2 DNA or 100 μg of B/NP DNA (control), i.m., 50 μl in each quadriceps. Mice received a boost with rAd four weeks later, 10^10^ total viral particles/mouse in 50 μl i.n. under isoflurane anesthesia. Some additional mice received rAd as sole immunization, or two rAd doses, one month apart, also given i.n. under anesthesia. For challenge, mice were infected i.n. under isoflurane anesthesia with A/FM-ma at indicated doses. Body weight and survival until weight loss endpoint were followed until surviving animals were recovering weight. After influenza virus challenge, the animals were observed twice daily including weekends (once by laboratory staff, once by vivarium staff). The animals were weighed once per day during this time. Deaths in groups that had not been challenged with influenza virus were very infrequent and occurred only in the elderly groups. Mice were euthanized if weight fell below 75% of individual starting weight, unless specified otherwise. The study was carried out in strict accordance with the recommendations in the Guide for the Care and Use of Laboratory Animals of the National Institutes of Health. This study was approved by the Institutional Animal Care and Use Committees at the Center for Biologics Evaluation and Research (CBER; Protocol #1991–06) and carried out in animal facilities accredited by the Association for Assessment and Accreditation of Laboratory Animal Care International. Experiments were performed according to institutional guidelines. Vaccination of different age groups in a figure was not always carried out simultaneously. When it was, that is indicated in the figure legends.

### Blood and Mucosal sampling

Mice were euthanized with an overdose of ketamine/xylazine corresponding to 300 mg/kg ketamine and 60 mg/kg Xylazine. BAL and lung cells were obtained as described [18]. Blood samples were collected from the abdominal vena cava or tail vein in BD Microtainer tubes (Franklin Lakes, NJ) and microcentrifuged 3 minutes at 13,200 g. Serum was heat-treated at 56°C for 30 minutes.

### Peptides and proteins

Peptides NP_147-155_ (TYQRTRALV), NP_55-69_ (RLIQNSLTIERMVLS), M2e_2-24_ consensus sequence (SLLTEVETPIRNEWGCRCNDSSD) of the highly conserved [11], surface-exposed M2 ectodomain (M2e), and SARS M_209-221_ (HAGSNDNIALLVQ) were synthesized by the CBER core facility. Recombinant nucleoprotein (rNP) from A/PR/8/34(H1N1) was purchased from Imgenex (San Diego, CA)

### T-cell ELISPOT

Lungs and spleens were harvested, processed, and interferon (IFN)-γ ELISPOT performed as described [23] with substrate from KPL (Gaithersburg, MD).

### Antibody analysis

Antibody levels were measured by enzyme-linked immunosorbent assay (ELISA) as described [13] using NUNC 96-well plates and human-adsorbed alkaline phosphatase-conjugated goat anti-mouse IgG or IgA (Southern Biotechnology Associates, Birmingham, AL). For measurement of IgG antibodies to adenovirus, pAd/CMV/V5-GW/lacZ was used to transfect 293 cells (ATCC, Manassas, VA), and a lysate prepared and clarified by centrifugation. A 1:200 dilution of clarified lysate in phosphate buffered saline (PBS) was used to coat ELISA plates. Absorbance at 405 nm was measured at 30 minutes in a Vmax kinetic microplate reader (Molecular Devices, Sunnyvale, CA).

### Flow cytometry

#### Memory marker staining

Lung T-cell phenotypes were assessed as described [19]. 2 x 106 lung cells/well were surface-stained with anti-CD3-eFluor450, anti-CD8-APC-Cy7, anti-CD62L-PE-Cy7, anti-CD69-PE, anti-CD127-PerCP-Cy5.5 (eBiosciences, San Diego, CA), and Live/Dead fixable green viability stain Vivid for 488 nm excitation (Invitrogen). The following tetramer was obtained through the NIH Tetramer Facility: NP_147–155_-H2-K^d^ Tetramer-APC (TYQRTRALV) (Atlanta, GA). 50,000 events per sample were acquired on an LSRII flow cytometer.

FACS Diva V6 software (BD Biosciences, San Jose, CA) was used for data acquisition and FlowJo V7.6.5 (TreeStar, Ashland, OR) for data analysis and display. Single color-stained cells were used for compensation, and fluorescence minus one (FMO) controls were used for gate setting.

#### CD107 staining

For stimulated cells, 106 cells/well were suspended in 100 μl MEM (Cellgro, Manassas, VA)/5% FBS medium containing anti-CD107a-eFluor450 (LAMP-1) (eBiosciences, San Diego, CA), 1 μg/mL each of anti-CD28 and -CD49d (BD Biosciences) and 0.7 μg/ mL monensin (BD Biosciences), with or without peptides: 2.5 μg/mL each of NP_147-155_, NP_55-69_, and M2e_2-24_ or 2.5 μg/mL SARS M for 5–6 hours. After incubation, cells were washed and stained with Live/Dead fixable blue viability stain Vivid for UV excitation (Invitrogen). Mouse CD16/32-specific monoclonal antibody 2.4G2 was added to cells before incubation with anti-CD3-PerCp-Cy5.5, anti-CD8-APC-Cy7, anti-CD4-AF700 (all from BD Biosciences), anti-CD107a, and NP147–155-H2-Kd Tetramer-APC. Following washing, the cells were fixed and permeabilized with Cytofix/Cytoperm buffer (BD Biosciences), and then incubated with anti-CD3, anti-CD8, anti-CD4 antibodies labeled as explained above. 30,000 events were acquired on a Fortessa flow cytometer (BD Biosciences,).

#### Cytotoxic assay

Cytotoxic activity was assessed by flow cytometry. As targets, naïve BALB/c splenocytes were labeled for an *ex vixo* cytotoxicity assay as described in [29] with either carboxyfluorescein diacetate succinimidyl ester (CFSE) or chloromethyl-benzoyl-amino-tetramethyl-rhodamine (CMTMR) (Molecular Probes, Eugene, OR). Cells labeled with CFSE were pulsed with a mixture of 1 mM each of NP_147-155_, NP_55-69_, and M2e_2-24_ peptides (target epitopes), or with SARS M peptide. Cells labelled with CMTMR were pulsed with SARS as a specificity control. Assays were performed in round-bottom 96-well plates with a 10:1 effector to target cell ratio. Lung lymphocytes from A/NP+M2 or B/NP immunized mice or mice infected with A/Mex under isoflurane were used as effectors. Cells were additionally stained with blue dead cell stain Vivid. After 5–6 hours in culture, 30,000 events were acquired on a Fortessa flow cytometer and analysis performed on gated live cells. The reduction in peptide-pulsed CFSE-labeled cells relative to the internal negative control of SARS-pulsed CMTMR-labeled cells served as a measure of specific cytotoxicity. SARS-pulsed CFSE-labeled cells were used to measure non-specific death. Percent specific cell death for both CFSE and CMTMR-stained cells is defined as 100 x [(% dead targets—% non-specific dead targets)/ (100%—non-specific dead targets)] with dead cells as defined by Vivid^+^ staining.

### Virus titration

Virus titers in lungs were determined by TCID_50_ as described [30], except that samples were homogenized using the PreCellys 24 system (Bertin Technologies; Atkinson, NH).

### Statistical analysis

GraphPad Prism 6.0 (GraphPad Software, San Diego, CA) was used for graphing and log-rank analysis, SigmaPlot12 (Systat Software Inc., San Jose, CA) for remaining statistics. Comparison of survival used log-rank (Mantel-Cox) testing. T cell responses to influenza peptides were first compared to SARS M responses by paired t-tests. For those responses differing from SARS M, comparison of multiple vaccination groups used one-way ANOVA followed by pairwise comparisons by Holm-Sidak testing or Mann-Whitney rank sum testing when normality or equal variance failed. Viral titers were compared by one-way ANOVA. When additional pilot groups were run in an experiment, although not shown, we included them in ANOVA to apply the penalty for more groups.

## Results

### Systemic and mucosal antibody responses decrease with age

In some previous work, we used three i.m. DNA doses followed by i.n. rAd boost. [18]. For the present study, a single DNA priming dose was chosen as a streamlined regimen for emergency use. In many of the immunization groups, we tested both the DNA prime-rAd-boost regimen and the rAd i.n. without DNA priming. DNA priming is not necessary for protection in young animals, but was included for comparison in parts of the present study in case the requirements for protection were different in mice of different ages.

We immunized mice of different ages to A/NP+M2 using DNA i.m. followed by A/NP+M2-rAd i.n. boost, or A/NP+M2-rAd i.n. alone. Controls received B/NP DNA i.m. with B/NP-rAd i.n. boost. For the rAd vaccines we chose the i.n. route, because this route is more effective for rAd immunization than i.m., whether for boosting or alone [18, 19].

**Fig 1:**
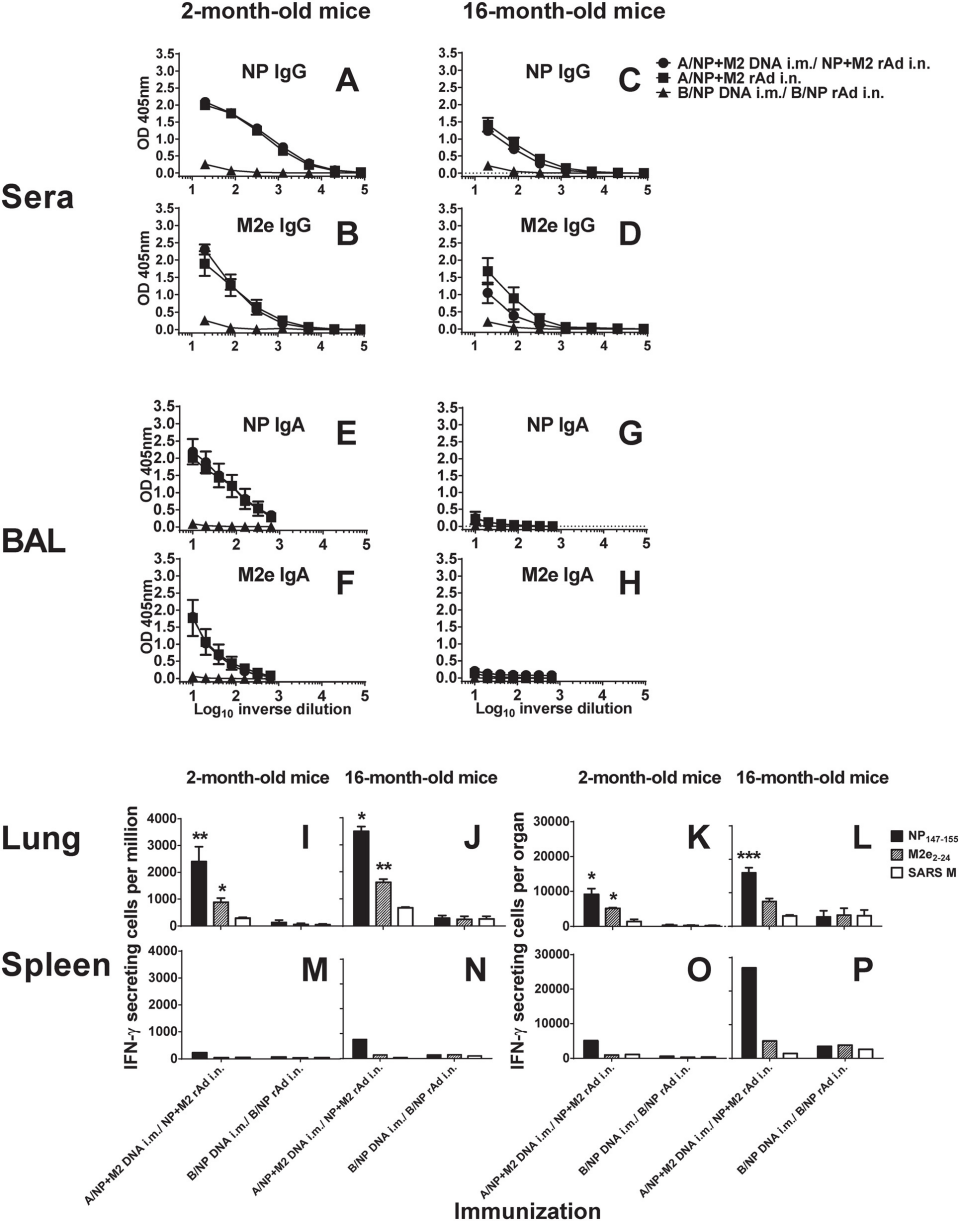
**Antibody responses in sera of young and middle-aged mice.** In all legends, ages given refer to age when immunization started. BALB/cAnNCr mice 2 months old (Fig 1A, 1B, 1E and 1F) or 16 months old (Fig 1C, 1D, 1G and 1H) were primed i.m. with 50 μg each of A/NP and A/M2 DNA or 100 μg of B/NP DNA. One month later these mice were boosted i.n. with 5×10^9^ particles each of A/NP-rAd and M2-rAd (solid circles), or 1×10^10^ particles of B/NP-rAd (solid triangles) respectively. A third group was given i.n. 5×10^9^ particles each of A/NP-rAd and A/M2-rAd without priming (solid squares, with rAd given at the same time point the other two groups were given the rAd boost). Sera and BAL were collected two to three weeks after the rAd immunization. ELISA testing of all serum samples, and of all BAL samples, was simultaneous. Antibody responses were measured by ELISA as described in Materials and Methods, on plates coated with NP or M2e as indicated. For serum (Fig 1A–1D), plates were developed with enzyme conjugates recognizing total IgG. For BAL (Fig 1E–1H), plates were developed with conjugates recognizing total IgA. Results shown are the mean absorbance ± SEM of 4 mice per group, except that only 3 BAL samples were available for the A/NP+M2 old-mice group. Background from secondary antibody alone was subtracted. **T cell responses in lungs and spleens of young and middle aged mice.** BALB/cAnNCr mice 2 months old (Fig 1I, 1K, 1M and 1O) or 16 months old (Fig 1J, 1L, 1N and 1P) were immunized as above. About 2 weeks after the rAd immunization, T cell responses were determined by IFN-γ ELISPOT. IFN-γ responses of lung cells from individual mice (I-L) or pooled spleen cells (M-P) are shown following stimulation with peptides NP_147–155_, M2_e2–24_, and SARS M as a specificity control. Bars show mean total lung IFN-γ secreting cells per million (I, J, M, and N) or per organ (K, L, O, and P) from 3 mice aged 2 months and 4 mice aged 16 months per group. Error bars indicate ± SEM in I, J, K, and L. Results were first compared pairwise by t-test to SARS M. For those differing significantly from SARS, immunization groups were compared to all other groups at the same time point using one way ANOVA followed by the pairwise Holm-Sidak method. Significant differences from the B/NP-rAd immunized control group are as follows: * p≤0.001 or ** p≤0.01 or ***p≤0.05.

IgG antibodies to NP and M2 were readily detectable in sera of young vaccinated mice (Fig 1A and 1B). Responses were at least as high in 11-month-olds as in two-month-olds (S1 Fig), but somewhat lower in 16-month-olds than two-month-olds (Fig 1C and 1D).

Immunized young mice had both IgG (S2A and S2B Fig) and IgA antibodies in BAL (Fig 1E and 1F), with a higher IgA response to NP than to M2e. In 16-month-old mice, BAL antibody responses were negligible (Figs 1G and 1H and S2C and S2D).

Responses were generally similar whether the mice had been given the DNA prime-rAd-boost regimen or only rAd.

### T-cell responses with age

**Fig 2:**
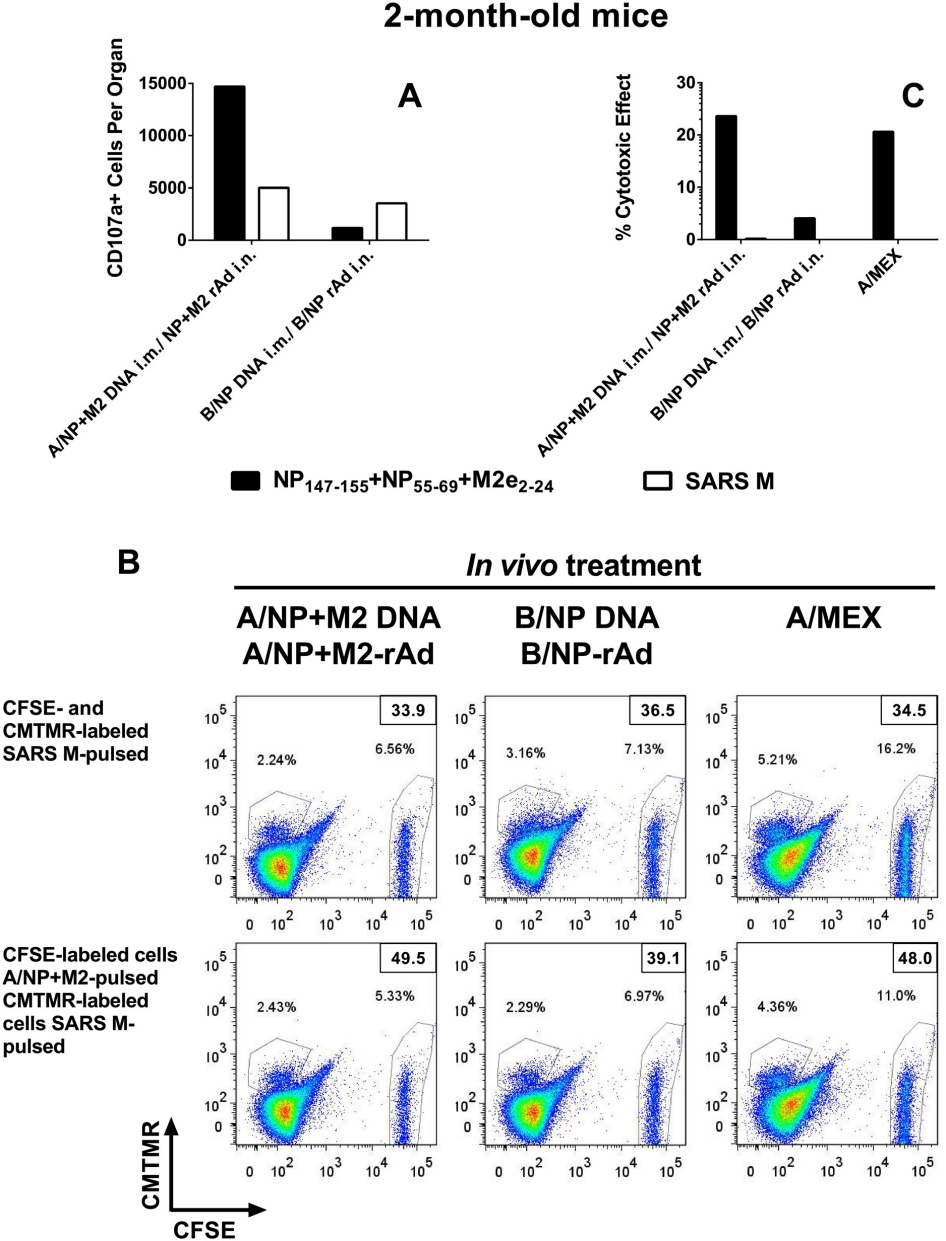
Cytotoxic activity. BALB/cBy mice of age 2 months were immunized as in Fig 1, and lung cells analyzed about 3 weeks after the last vaccination. Lung cells from mice infected 6 days earlier with A/Mex were used as a positive control for cytotoxicity. (A) Total number of CD3^+^CD8^+^CD107^+^Tetramer^+^ cells in lung cells pooled from 4 animals was determined by multicolor flow cytometry after stimulation with peptides NP_147–155_+NP_55–69_+M2_e2–24_ (NP/M2e; solid bars), or SARS M peptide control (open bars). (B) Splenocytes were labeled with CFSE or CMTMR, and pulsed with peptides as indicated. 200,000 of these splenocytes were then incubated with 2x10^6^ lung cells of A/NP+M2- or B/NP-immune or A/Mex-infected mice for 5.5 hours. Staining with CFSE is displayed on the horizontal axis, staining with CMTMR on the vertical axis. Dead cells were defined as Vivid^+^ and values shown in the top right corner for each treatment. Top panel: Both CFSE- and CMTMR-labeled cells were control-pulsed (SARS M peptides). Bottom panel: CFSE-labeled target cells were pulsed with NP/M2e peptides, CMTMR-labeled target cells were control-pulsed. (C) Percent specific cell death calculated from the flow cytometry data as described in Materials and Methods. All results shown are representative of two experiments. We were not able to obtain enough elderly mice for comparison of CTL activity to that in young mice.

Vaccination to A/NP+M2 by regimens including rAd has previously been shown to induce lung T-cell IFN-γ ELISPOT responses [18, 19]. As shown in Fig 2, it can also induce cytotoxic activity in the lungs and up-regulate surface expression of CD107a (a CTL degranulation marker [31]). For study of functional T-cell responses in DNA prime-rAd-boost mice of different ages, IFN-γ ELISPOT was used to analyze cells collected 2–3 weeks after the rAd boost. Restimulation used NP_147-155_ (an immunodominant H-2K^d^-restricted epitope) [32], M2e_2-24_ (MHC class II restricted epitope [33]), and SARS (specificity control) peptides. A/NP+M2 DNA prime-rAd-boost vaccination induced lung T-cell responses to NP_147-155_ significantly above B/NP-immunized controls at both two and 16 months (Fig 1I–1L). Responses to the MHC class II restricted epitope M2e_2-24_ [33] were specific but modest (Fig 1I–1K).

Splenic responses differed from those in lungs, as previously observed [18]. Vaccination i.n. produced low frequencies of NP_147-155_ specific T-cells (Fig 1M and 1N). When recalculated as responding T cells/organ (Fig 1K, 1L, 1O and 1P), responses were on average >5 times higher in 16-month-old animals than younger ones for the dominant epitope NP_147-155_ (Fig 1O and 1P). The difference in T cell response was less dramatic in lungs.

**Fig 3:**
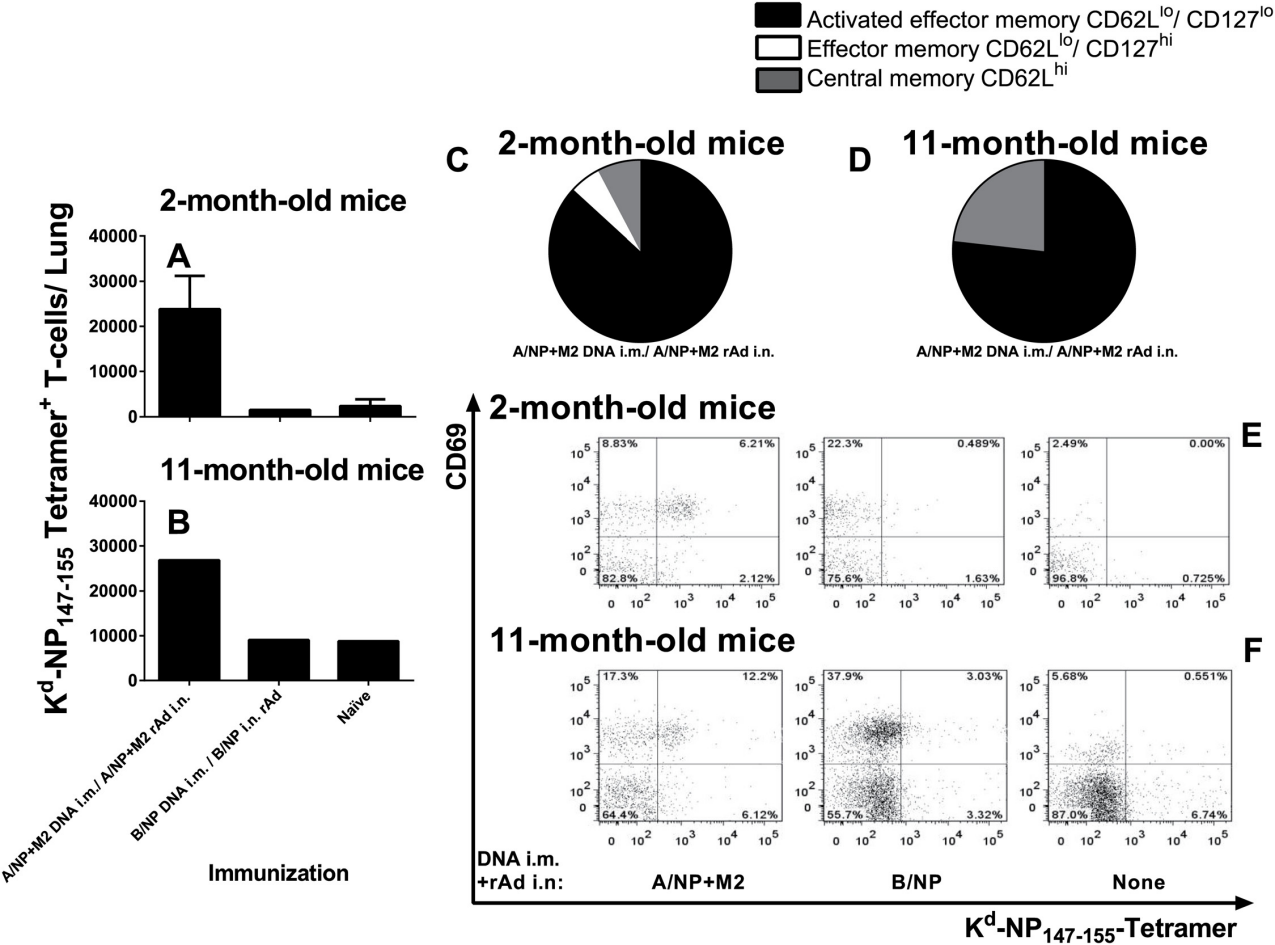
Characterization of lung T cells from young and middle-aged mice. BALB/cAnNCr mice were immunized at 2 months (A, C, and E) or 11 months old (B, D, and F) as in Fig 1 or left unvaccinated (naïve). Lung T cells were prepared and analyzed by flow cytometry. (A, B) Total numbers of K^d^-NP_147–155_-tetramer^+^ CD8^+^ T cells recovered from the lungs 12–13 weeks after vaccination were determined by multicolor flow cytometry. Bars show mean ± SEM of 3–4 animals per group for the two-month-old mice (A). For 11- month-old mice, cell yield after Ficoll was low and lung cells had to be pooled to permit analysis (B). (C, D) Lung CD8^+^ T cell phenotypic analysis: Proportions of activated effector memory (CD62L^lo^, CD127^lo^), effector memory (CD62L^lo^, CD127^hi^) and central memory (CD62L^hi^) among tetramer^+^ CD8^+^ T cells from A/NP+M2 vaccinated animals are displayed as pie charts. (E, F) Activation status of pooled lung CD8^+^ T cells characterized by positive staining for both tetramer and CD69 marker. Fluorescence minus one (FMO) controls were used to identify gating boundaries and adjusted based on internal negative controls for each treatment group. Percent of total CD8^+^ T cells are displayed per quadrant.

Further characterization of responding T cells from DNA prime-rAd-boost immunized mice revealed large numbers of NP_147-155_ tetramer^+^ CD8^+^ T-cells seen in the lungs of two- and 11-month-old mice (Fig 3A and 3B). Most had a CD62L^lo^, CD127^lo^ (activated) phenotype, with fewer CD62L^hi^ (central memory) cells (Fig 3C and 3D). Few cells from young animals and none from 11-month-olds possessed a CD62L^lo^, CD127^hi^ (effector memory) phenotype (Fig 3C and 3D). Compared to controls, animals immunized to A/NP+M2 had more influenza-specific cells expressing the early activation marker CD69 (Fig 3E and 3F).

### Protection from high-dose H1N1 challenge

**Fig 4:**
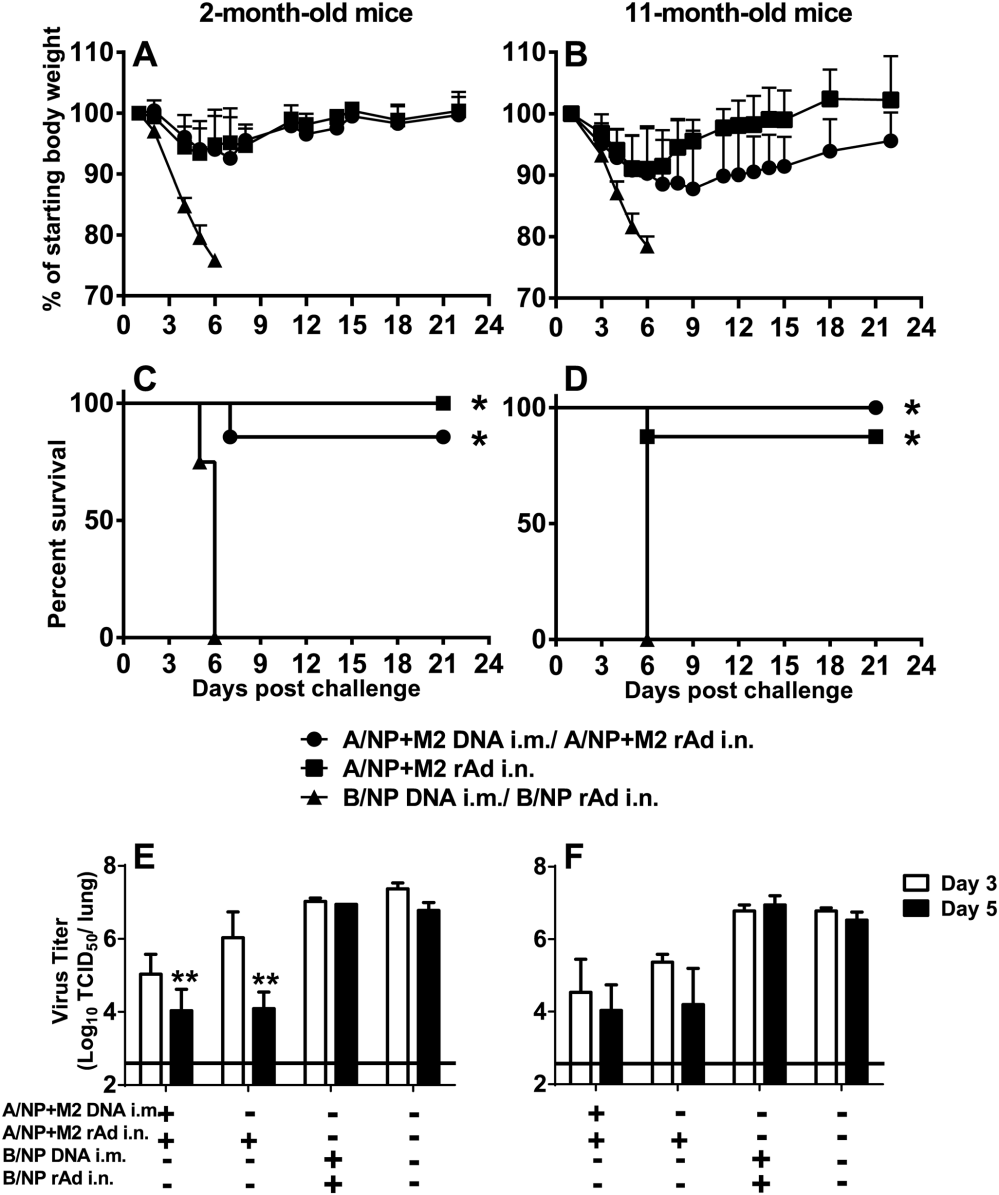
Morbidity, survival, and lung viral titers after influenza virus challenge of young and middle-aged mice. Groups of 14 BALB/cAnNCr mice of age 2 months (A, C, and E) or 11 months (B, D, and F) were immunized as in Fig 1. In addition, 6 mice per age group were left unvaccinated (naïve, Fig 4E and 4F). One month after rAd, animals were challenged with 10^4^ TCID_50_ A/FM-ma. Panels A and B show the mean % starting body weight + SD of 8 mice per group. Panels C and D show survival until weight loss endpoint; *p≤0.001 by the log-rank (Mantel-Cox) for all groups compared to B/NP control. Lung virus titers (E and F) were determined by TCID_50_ assay on the lungs of 3 animals per group harvested on day 3 (open bars) and day 5 (solid bars) after challenge, along with lungs from naïve mice of the same age infected at the same time. Shown are the mean ± SEM of 3 mice per group with the limit of detection indicated by a horizontal line. ** p≤0.01 was determined by ANOVA vs. the B/NP and naïve control groups. The difference was statistically significant (**p<0.01) on day 5 for two-month-old animals. The 2- and 11-month-old groups were not performed simultaneously, so we cannot conclude that there was superior control of viral replication in one age group.

A month after rAd immunization, mice were challenged with 10^4^ TCID_50_ of A/FM-ma. Both DNA prime-rAd-boost and i.n. rAd only vaccination to A/NP+M2 was highly protective in young and middle-aged mice, reducing weight loss (Fig 4A and 4B) and preventing most mice from reaching the weight loss endpoint, while B/NP controls succumbed (survival p<0.001 for both ages compared to B/NP-immunized controls, Fig 4C and 4D). Older animals lost somewhat more weight than younger ones, and recovered it more slowly (Fig 4A and 4B).

We tested lung virus titers after challenge to assess immune-mediated control of virus replication (Fig 4E and 4F). Mice immunized to A/NP+M2 with either DNA prime-rAd-boost or rAd alone showed lower viral titers than B/NP and naïve groups as early as day 3 post-challenge.

### Immune responses in elderly mice

**Fig 5:**
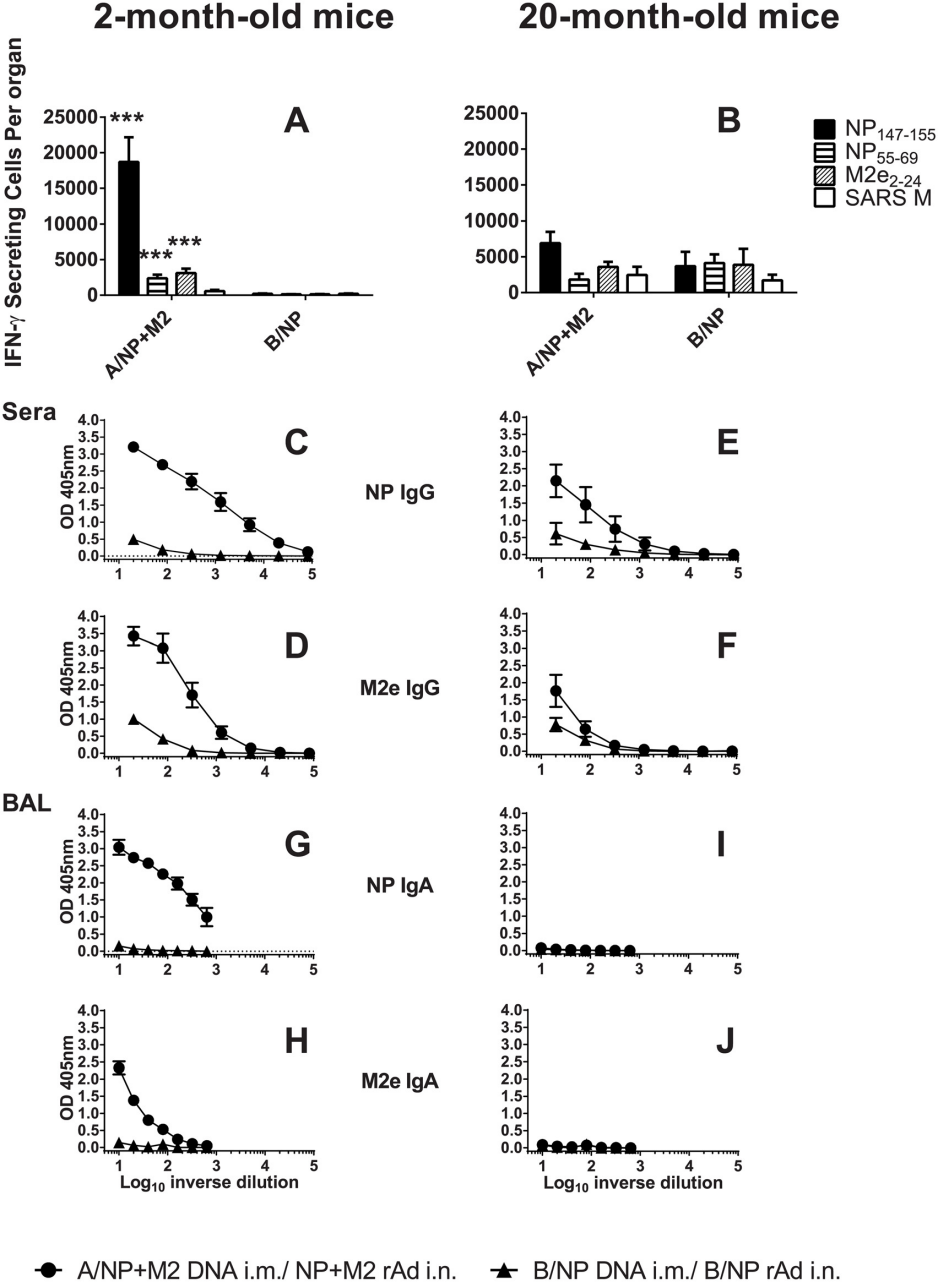
Immune responses in young and elderly mice. BALB/cBy mice of age 2 months (A, C, D, G, and H) or 20 months (B, E, F, I, and J) were immunized as in Fig 1. **T cell responses:** Three weeks after the rAd immunization, T cell responses in lungs were determined by IFN-γ ELISPOT (A, B). IFN-γ responses of lung T cells are shown following stimulation with peptides NP_147–155_, NP_55–69_, and M2_e2–24_. SARS M stimulated cells were used as controls. Bars show mean total lung IFN-γ secreting cell number per organ ± SEM for 3 or 4 mice per group. ***p≤0.05 comparison of A/NP+M2 versus B/NP by Mann-Whitney rank sum test determined for each peptide, performed only for those samples significantly above SARS M. **Antibody responses:** (C-J) Samples were collected three weeks after rAd from A/NP+M2 DNA prime-rAd boost (solid circles) and B/NP DNA prime-rAd boost (solid triangles) mice. Sera (C-F) or BAL (G-J) were analyzed by ELISA. Results show the mean of absorbance ± SEM of sera from 3–12 mice or BAL samples from 3–4 mice per group. Background from secondary antibody alone was subtracted.

We extended the study to an age near the limit of the mouse lifespan. Due to difficulty in obtaining mice 20 months old, we used BALB/c mice from a different source and subline, and available numbers were limited. First, we verified that young animals from this source and subline responded to vaccination similarly to those in previous experiments. Young BALB/cBy animals vaccinated to A/NP+M2 by DNA prime-rAd boost had robust IFN–γ T-cell ELISPOT responses (Fig 5A). Responses to NP_147-155_, NP_55-69_ (T helper epitope [34]), and M2e_2-24_ peptide were significantly above the B/NP group (p<0.05). In 20-month-old animals (Fig 5B), responses were much weaker, not reaching statistical significance for any peptide above the higher background.

In elderly mice, serum IgG responses to NP were weaker compared to young mice (Fig 5C vs. 5E), both in maximum binding and in titer. Serum responses to M2e were strong in young mice, but very weak in elderly mice (Fig 5D vs. 5F).

In BAL, young mice had substantial levels of IgA antibodies, with greater levels against NP (Fig 5G) than M2e (Fig 5H). In contrast, 20-month-olds had negligible responses (Fig 5I and 5J).

### Protection of elderly mice from H1N1 challenge

**Fig 6:**
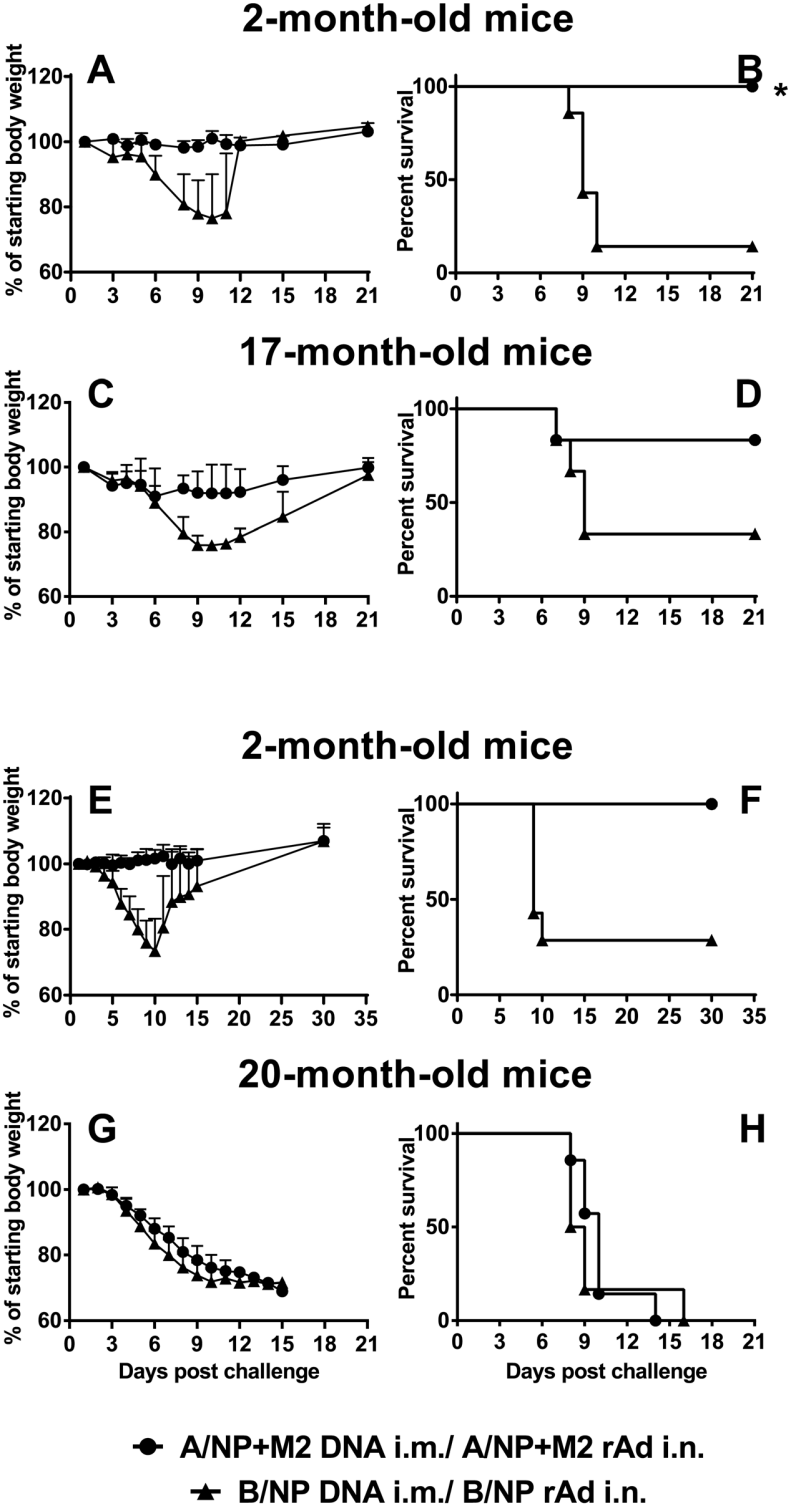
Morbidity and survival following challenge in young and elderly mice. (A-D) Groups of BALB/cAnNCr mice or (E-H) BALB/cJ mice were immunized to A/NP+M2 (N = 6 to 8) or B/NP (N = 6 to 7) by DNA priming followed by rAd boosting. They were challenged with 200 TCID_50_ of A/FM-ma one month after rAd. (A, C, E, and G) Weight loss after challenge shown as the mean + SD. (B, D, F, and H) Survival until weight loss endpoint after challenge. Animals were euthanized if weight decreased by more than 30% of the individual starting weight (a pre-determined endpoint for this experiment so that even weak protection would have been seen in 20-month-old mice, if present). Not all groups were vaccinated and challenged simultaneously, but each old group was vaccinated and challenged simultaneously with a young control group of the same BALB/c subline. Young animals in different groups showed similar results.

A month after DNA prime-rAd boost immunization, mice were challenged with 200 TCID_50_ of A/FM-ma, a modest dose chosen to reveal protective immunity in elderly mice, if present. The control groups of young A/NP+M2-vaccinated mice lost little weight (Fig 6A and 6E) and all of them survived (Fig 6B and 6F). A/NP+M2 vaccination also protected 17-month-old mice, reducing morbidity (Fig 6C) and enhancing survival (Fig 6D). However, A/NP+M2 vaccination did not protect elderly animals of 20 months, which lost weight rapidly (Fig 6G) and reached the weight loss endpoint (Fig 6H). Controls of each age immunized against B/NP lost weight rapidly and most reached the weight loss endpoint (Fig 6A–6H).

**Fig 7:**
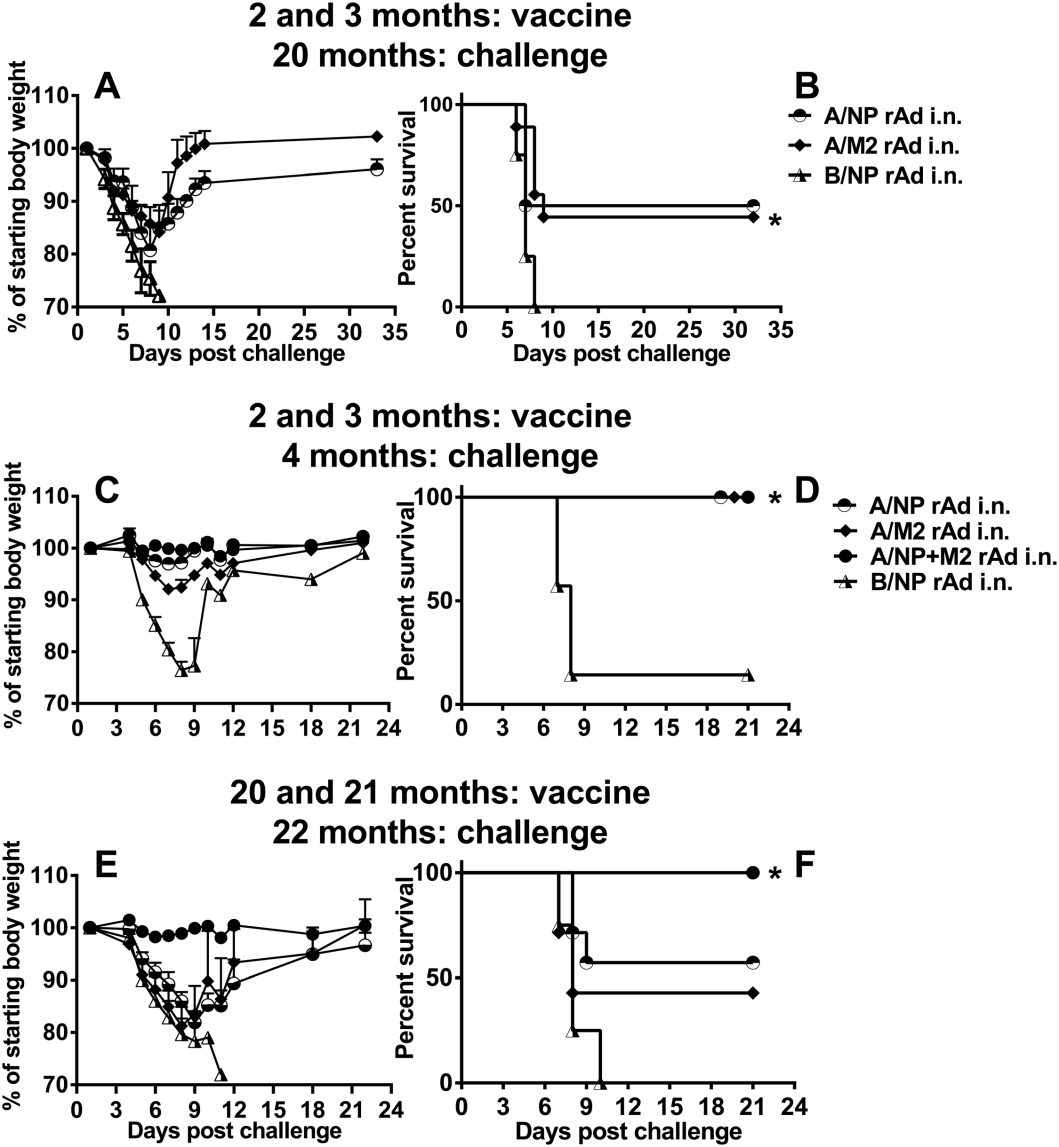
Morbidity and survival following rAd given twice and the impact of prior vaccination. (A-B) Groups of 4 (A/NP) or 9 (A/M2) or 4 (B/NP) BALB/cAnNCr mice were available from groups given 1×10^10^ particles of the respective rAds at two and three months of age. They were then challenged with 10^3^ TCID_50_ of A/FM-ma at 20 months of age. (A) Weight loss after challenge, shown as the mean + SD. (B) Survival after challenge until weight loss endpoint, p = 0.016 for A/M2 vs B/NP control group (*). The A/NP group was too small to reach significance for protection versus the B/NP control group. (C-F) Groups of BALB/cAnNCr mice (N = 4 to 7) were given 1×10^10^ particles of individual rAd, or 5×10^9^ of each rAd in the mixture of A/NP and M2 at two and three months of age (Fig 7C-D) or 20 and 21 months (Fig 7E and 7F), then challenged with 10^3^ TCID_50_ of A/FM-ma a month after the boost. (C, E) Weight loss after challenge shown as the mean + SD. (D, F) Survival after challenge until weight loss endpoint.

Immunological experience early in life can affect outcomes during old age. In an earlier study, mice had impaired influenza virus-specific CTL responses if primed when old, but better responses if primed when young and tested when old [35]. Early priming with influenza virus was also reported to lead to long-term maintanance of memory T cells [36]. We explored whether A/NP+M2 vaccination, which protects in part via T-cell activity [23, 24], might show a similar impact of priming early in life. We had mice vaccinated i.n. twice at two and three months of age with A/NP-rAd or A/M2-rAd, or B/NP-rAd, and held them until they reached 20 months of age. We challenged these animals with 10^3^ TCID_50_ A/FM-ma to test for long-term protection provided by the early vaccination (Fig 7A and 7B). The mice immunized to A/NP or A/M2 lost less weight than controls (Fig 7A) and approximately half of them survived (Fig 7B). B/NP-vaccinated controls all reached the weight loss endpoint. This result suggests that a universal vaccine, even one containing a single antigen, can provide some long-term protection extending into old age.

We then considered the possibility that a rAd boost might strengthen protection in the elderly. Elderly mice were immunized with rAd i.n. twice at an interval of one month. Groups were given A/NP-rAd or A/M2-rAd, or A/NP+M2 rAd, or B/NP-rAd. Two month-old mice were used as controls. Antibody responses were measured in these groups of mice. Serum IgG responses to NP and M2e in old mice were somewhat weaker than in young mice; there was some increase in antibody after the boost (S3 Fig). We also measured serum IgG responses to adenovirus (S4 Fig). Responses were minimal after priming in both age groups. They increased after the boost, more so in young mice than in old. These groups of mice were challenged one month after the second rAd dose with 10^3^ TCID_50_ A/FM-ma (Fig 7C–7F). The young mice immunized to A/NP or A/M2 or A/NP+M2 lost less weight than controls (Fig 7C), with vaccination to A/NP+M2 giving the best protection as we have seen before [13], and all three of the vaccine groups survived (Fig 7D). Weight loss was more severe in the elderly mice (Fig 7E), but approximately 43% of the A/M2-rAd-, 57% of the A/NP-rAd- and all of the A/NP+M2-rAd-vaccinated mice survived (Fig 7F). B/NP-vaccinated controls all reached the weight loss endpoint. These results are evidence that universal vaccination of this type can provide some protection to elderly recipients if they are given two doses.

## Discussion

Given the dramatic differences in influenza risk with age, it is important to study new candidate vaccines for their potential to protect different age groups. Alternative adjuvants, doses, and routes of administration are being explored to overcome limitations in effectiveness (reviewed in [37]). We tested a candidate universal influenza vaccine in mice of different ages, both for immune responses and for protection against challenge infection.

Mice two and 11 months old responded with systemic and mucosal antibodies, as well as with influenza virus-specific memory T cells in the lungs demonstrated by tetramer analysis. Analysis of surface markers showed high levels of activated effector memory T cells in the lungs of mice immunized at 2 months and 11 months. Moreover, vaccination of young mice increased IFN-γ secreting cells as measured by ELISPOT, CD107a expression, and influenza virus-specific cytotoxic activity of lung cells. Substantial T cell responses to A/NP+M2 were observed by ELISPOT even in mice 16 months of age, but antibody responses declined by this age, especially in the mucosal compartment. By 20 months of age, mice showed greatly impaired cellular and humoral immune responses to vaccination. The limited availability of elderly animals for purchase and attrition in groups we aged in our facility meant we could not perform all experiment types with mice 20 months old. However, T cell ELISPOT responses at this age were marginal. Antibody responses were detectable but lower in sera than in young mice, and negligible in BAL.

Immunization with A/NP+M2-rAd given once protected young and middle-aged mice up to 17 months of age against challenge with virulent mouse-adapted A/FM-ma. B/NP-immunized controls were not protected, as all of them reached the weight loss endpoint, confirming that protection is antigen-specific.

Both antibodies and T cell immunity may contribute to protection. While antibodies to NP and M2 are not neutralizing, they can be protective [11, 23, 38]. The mechanism could be antibody-dependent cell-mediated cytotoxicity (ADCC) [39] and perhaps also intracellular disruption of viral replication, as seen in a variety of other examples reviewed in [40].

In mice, passively transferred A/M2-specific antibody reduced influenza A [23, 41], but not influenza B [41], virus replication. Pre-existing immune responses including M2- and NP-specific antibodies in BAL and lung-resident virus-specific T cells with an activated effector memory phenotype, are likely to be important for early control of infection at the respiratory mucosa. Crucially, unlike naïve T cells, effector memory T-cells can immediately exert antiviral functions, including secretion of perforin, granzymes, and cytokines such as IFN-γ, upon encountering the challenge virus. While cytotoxic activity is generally thought to be protective, some argue that CD8^+^ T cell stimulation might exacerbate disease, as shown in old mice for one regimen [42]. We saw no evidence of exacerbated morbidity (weight loss) with A/NP+M2 vaccine compared to control.

Both mice and humans show impaired T cell responses with age. Age-related changes in protein phosphorylation involved in T lymphocyte proliferation have been reported [43], and responses to mitogens are blunted in old compared to young mice [43]. Following influenza virus infection of immunized mice, *in vitro* restimulated T cell responses are delayed and decreased in old compared to young mice [44, 45]. In humans, immune responses are greatly affected by aging, with decreased antibody responses and lower T cell proliferation and cytotoxicity [7, 46–49], which affect recovery from infection (reviewed in [50]). The elderly also possess a reduced proportion of naïve cells and decreased repertoire diversity [51], thus decreasing their potential to respond to new antigens.

Although their response to new antigens is impaired, cytotoxic T cell responses in old mice are greatly enhanced if the mice had been primed while young [35]. We explored the implications of this for vaccination with A/NP-rAd or M2-rAd given twice in youth. Despite the 16-month gap between vaccination and challenge at 20 months, partial protection was seen. We had shown previously that a single vaccination to A/NP+M2 in young mice provided protection against virulent challenge 8–10 months later [18, 19], but this is our first demonstration of protection in elderly mice.

In the hope of developing a regimen effective when begun in old age, we examined a regimen using two doses of rAd. This regimen was successful in protecting elderly mice. The improvement in protection compared to a single dose may be due to the boosting, since antibody responses to NP and M2 rose after the second dose (S3 Fig) Alternatively, improved protection may be due to response kinetics, with the delay in challenge providing a longer time interval for immune response maturation.

A potential drawback to this two-dose rAd regimen is that prior exposure to Ad of the same serotype could interfere with vaccine effectiveness, but use of the intranasal route has been reported to circumvent prior anti-Ad immunity to a considerable extent [52, 53]. Indeed, in our system, the antibody response to adenovirus was marginal after one i.n. dose and considerable only after the boost (S4 Fig). Thus blocking might not be induced by a single dose. Humans have blocking antibody from a different source, namely prior wild type adenovirus infections. However, alternative vectors such as those based on Pan Adenovirus type 3 [54, 55] or other nonhuman primate adenovirus vectors [56] are being developed and are not neutralized by antibodies widely circulating in the human population [55].

We and others have discussed previously the protective value of heterosubtypic immunity (reviewed in [9]) and others have emphasized the potential downside of annual strain-matched vaccination, which can provide sterilizing immunity but thereby preclude beneficial T cell priming induced by infection [57–59]. The universal vaccine studied here would induce broad cross-protective immunity, including T cell priming, without the health risks of undergoing a wild type influenza virus infection. Critically, elderly humans have been primed by previous infections, asymptomatic exposures, and/or live-attenuated influenza vaccines, and may thus respond to A/NP+M2 vaccination more strongly than elderly SPF mice that have never had such previous exposures.

Our findings suggest advantages to offering immunization while the immune system is young and optimally responsive in order to protect later in life. Vaccination during youth to antigens conserved in all influenza viruses might not only enhance function at the time, but have a lasting impact by stimulating relevant repertoire elements while they are still available and thus preserving responsiveness. In addition, the findings suggest some promise for first use of the universal vaccine in the elderly, if given with an enhanced regimen. Overall, the work helps define strategies to improve vaccine effectiveness in the elderly and thus has important public health implications.

## Supporting Information

S1 Fig Antibody responses in sera from another middle-age comparison. As Fig 1 but BALB/cAnNCr mice were two months (A and B) or 11 months (C and D) old. Analyses were performed at two to three weeks after the boost. (A, C) NP-specific IgG; (B, D), M2e-specific IgG. Responses in serum were measured by ELISA as described in Materials and Methods. Results are shown for 6 mice per group. Background from secondary antibody alone was subtracted. ELISA testing of all samples was simultaneous.
(EPS)

S2 Fig IgG antibody responses in BAL of young and middle-aged mice. BALB/cAnNCr mice 2 months old (A and B) or 16 months old (C and D) were immunized as in Fig 1. Samples were collected two to three weeks after the rAd immunization. Antibody responses in BAL were measured by ELISA as described in Materials and Methods on plates coated with NP or M2e as indicated, and developed with enzyme conjugates recognizing total IgG. Results shown are the mean absorbance ± SEM of 4 mice per group, except for the A/NP+M2 primed group where only 3 mice were available. Background from secondary antibody alone was subtracted. ELISA testing of all samples was simultaneous.
(EPS)

S3 Fig IgG responses to NP and M2e in sera after one and two adenovirus doses. NP and M2e-specific IgG in 2-month-old mice (A, B, E, and F) or 20-month-old mice (C, D, G, and H). Responses in serum were measured by ELISA as described in Materials and Methods except that plates were coated with rNP from GenScript (Piscataway, NJ). Results are shown for 4 mice per group after the first (A, C, E, and G) and second (B, D, F, and H) doses of rAd. Background from secondary antibody alone was subtracted. ELISA testing of all samples was simultaneous.
(EPS)

S4 Fig IgG responses to adenovirus in sera after one and two adenovirus doses. Adenovirus-specific IgG in 2-month-old mice (A and C) or 20-month-old mice (B and D). Responses in serum were measured by ELISA as described in Materials and Methods after the first (A and B) and second (C and D) doses of rAd. Results are shown for 4 mice per group. Background from secondary antibody alone was subtracted. ELISA testing of all samples was simultaneous.
(EPS)
